# Antibiotic-impregnated cement spacer as definitive management for osteomyelitis

**DOI:** 10.1186/s12891-015-0704-1

**Published:** 2015-09-14

**Authors:** Xu-Sheng Qiu, Xin Zheng, Hong-fei Shi, Yan-cheng Zhu, Xia Guo, Hai-jun Mao, Guang-yue Xu, Yi-xin Chen

**Affiliations:** Department of Orthopaedics, Drum Tower Hospital, the Affiliated Hospital of Nanjing University Medical School, No. 321 Zhongshan Road, Nanjing, China

## Abstract

**Background:**

Osteomyelitis is a challenge for orthopaedic surgeons. There is a lack of scientific evidence to guide treatment. The purpose of this study was to report the clinical outcome of unplanned retention of antibiotic-impregnated cement spacer (ACS) in the management of osteomyelitis.

**Methods:**

Eight patients (7 with tibial infections and 1 with a calcaneal infection) with osteomyelitis received radical debridement and insertion of an ACS into the bone defect as the definitive management. The mean follow-up period was 2 years (6 months to 4 years). All of these patients had a cement spacer in place.

**Results:**

No patient exhibited radiographic evidence of excessive bone loss. The patients reported no or occasional mild pain and exhibited complete weight-bearing abilities, with the exception of one patient who required a crutch because of a spinal cord injury. Signs of recurrence of the osteomyelitis were not noted in any of the patients, and no fractures occurred at last follow-up.

**Conclusion:**

Our study suggests that a proportion of patients with unplanned retention of ACS appear to function well without necessarily requiring further surgical intervention.

## Background

Osteomyelitis is a challenge for orthopaedic surgeons. The management of osteomyelitis has received minimal attention, and there is a lack of scientific evidence to guide treatment. Basically, a palliative or a curative approach must be decided. Interdisciplinary treatment with close collaboration between trauma surgeons, plastic surgeons, anaesthetists, microbiologists and radiologists is essential for successful management of osteomyelitis [1]. Curative management of osteomyelitis requires aggressive surgical debridement and reconstruction followed by antibiotic therapy. Soft tissue coverage and dead space management after extensive debridement is paramount because dead spaces may contribute to infection recurrence. All efforts should be made to optimize the host prior to treatment, such as smoking cessation and close control of blood glucose in patients with diabetes mellitus [2, 3].

If a curative approach is chosen, most of the patients with osteomyelitis receive two-stage management in our centre. The first stage includes radical debridement and insertion of an antibiotic-impregnated cement spacer (ACS) into the bone defect. The bone is stabilized, and soft tissue is repaired if needed. The second stage is performed 6 to 8 weeks later. The spacer is removed; a cancellous autograft is placed within the bone defect.

During our practice, eight patients with osteomyelitis received only first-stage treatment due refusal of second-stage treatment or patients were medically unfit without major complications. The possibility of ACS as definitive management for osteomyelitis is promising.

Although reports have been published on the long-term use of ACS in infected total hip replacements [4, 5], total knee replacements [6], total shoulder replacements [7, 8], total ankle replacements [9] and diabetic feet [10, 11], similar studies are not available on the use of ACS as a permanent solution for osteomyelitis. Therefore, the purpose of this study was to evaluate the outcome of using ACS as a definitive management for osteomyelitis.

## Methods

Between January 2011 and June 2014, a total of 38 patients with osteomyelitis were treated at our institution using the described technique. Eight of the 38 patients (six men, two women) received only the first-stage treatment. These patients did not receive the second-stage procedure because they were either medically unfit (*n* = 2) or refused revision surgery (*n* = 6). Of these patients, 7 had tibial infections (4 proximal, 1 shaft, and 2 distal), and 1 had a calcaneal infection. The patients underwent an average of 2 (range, 0 to 4) operations before referral to our centre. The delay between the occurrence of the bone infection and the treatment of bone infection at our institution ranged from 10 to 700 days (mean, 120 days). Detail patient information based on the “Seven-Item Comprehensive Classification System” for osteomyelitis is presented in Table 1 [12]. All patients were treated by the senior author (CYX). The mean age at the time of the first stage of reconstruction was 40.5 (range, 25 to 70) years.

**Table 1:** Detailed patient information according to the “Seven-Item Comprehensive Classification System” for osteomyelitis

Cases	Clinical presentation	Aetiopathogenesis	Anatomical pathology	Host type/age	Microorganism	Bone defect	Soft tissue defect
1	Delayed	Temporary implant (type 2)	Long bone (Stage 3)	Aa	Gram+	1	2 (1 cm^2^)
2	Chronic	Trauma	Long bone (Stage 3)	Ba	Mixed flora	1	0
3	Subacute	Haematogenous	Long bone (Stage 3)	Ba	Gram-	1	0
4	Chronic	Trauma	Long bone (Stage 3)	Aa	Negative	1	1 (2 cm^2^)
5	Acute	Trauma	Foot	Ba	Gram+	1	1 (1 cm^2^)
6	Chronic	Trauma	Long bone (Stage 3)	Aa	Mixed flora	1	1 (4 cm^2^)
7	Chronic	Trauma	Long bone (Stage 3)	Aa	Gram-	1	0
8	Chronic	Trauma	Long bone (Stage 3)	Aa	Gram+	1	0

**Fig. 1:**
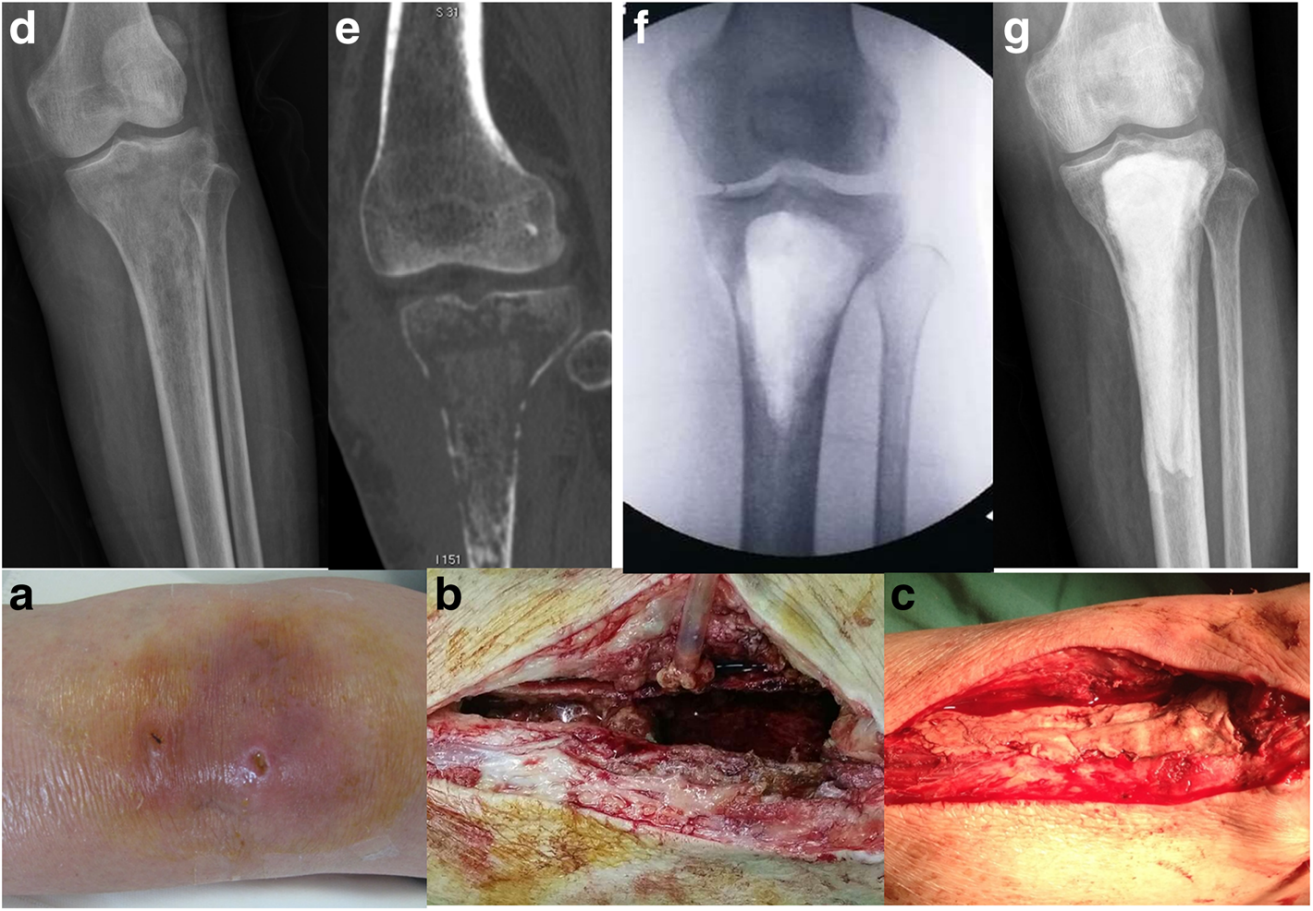
Case 3 (female, 70 years old) required long-term glucocorticoid treatment because of interstitial pneumonia. She suffered from left tibia osteomyelitis and failed treatment with intravenous antibiotic therapy. **a** Tenderness, warmth and effusion were observed on admission one month after the onset of osteomyelitis. **d**-**e** X-ray film and CT scan revealed bone destruction on admission. **b**, **f** hrough debridement was performed; a significant bone defect occurred after debridement. **c**, **g** The bone defect was filled with bone cement

Preoperative diagnosis of infection was made according to clinical, laboratory, imaging, microbiological, and pathohistological features. A history of sudden onset of pain, swelling, or wound drainage with or without fever and clinical findings of tenderness, warmth and effusion were indicative of infection (Fig. 1). Laboratory tests included white blood cell counts, erythrocyte sedimentation rate (ESR), and C-reactive protein (CRP) levels. An ESR of greater than 15 mm/h together with a CRP of greater than 8 mg/L is suggestive of infection. When an effusion was palpable, aspiration under sterile conditions was performed. The fluid was then sent for culture to aid in making a definitive diagnosis as well as deciding on the appropriate antibiotic to mix with the cement at the time of surgery.

Initially, all eight patients planned to receive a two-stage treatment. The first stage involved treating the bone infection, and the second stage involved reconstructing the bone defect. Initially, any internal bone fixations were removed. Deep tissue samples were obtained for microbiological analysis. After thorough soft tissue and bone debridement, all patients’ bones were relatively stable, and no stabilization with external fixators or other device was needed. The bone defect was then filled with antibiotic cement spacer (Fig. 1). Polymethyl methacrylate (PMMA) bone cement (Smith & Nephew, TN, USA) was used. We added additional sensitive antibiotics to the powder before mixing the powder and liquid (Table 2). If no culture was available, one gram of vancomycin and one gram of gentamycin were mixed into the cement (40 g). The volume of the bone defect was estimated from the volume of filled cement spacer. After thorough debridement, two patients needed soft tissue reconstruction. Our plastic surgery colleagues assisted with those cases requiring cutaneous/fasciocutaneous flaps for soft-tissue coverage.

**Table 2:** Culture results and local antibiotics used

Cases	Microorganism	Local antibiotics/40 g cement
1	Staphylococcus aureus	1 g vancomycin
2	Enterobacter cloacae, Enterococcus avium	1 g vancomycin/1 g imipenem
3	Escherichia coli	1 g imipenem
4	-	1 g vancomycin/1 g gentamycin
5	Staphylococcus aureus	1 g vancomycin
6	Staphylococcus aureus, Pseudomonas aeruginosa	1 g vancomycin/1 g imipenem
7	Acinetobacter baumannii	1 g imipenem
8	Staphylococcus epidermidis	1 g vancomycin

Postoperatively, patients were initially treated with sensitive antibiotics intravenously according to previous culture result. Then, the antibiotics were adjusted according to the deep tissue culture results. If the culture was negative, the patient received vancomycin intravenously. Antibiotic treatment lasted until CRP was less than 8 mg/L and ESR was less than 15 mm/h. The patients were allowed full weight-bearing movement once the wound was healed. All patients were evaluated postoperatively every 1 to 2 months.

After resolution of the infection, the patients in this study were either medically unfit (*n* = 2) or refused revision surgery (*n* = 6). Thus, the cement spacer was used as a definitive procedure.

All patients provided informed consent for inclusion in the study and consent was obtained from Case 3 patient for publication of individual information and accompanying images. The study was authorized by the Medical Ethics Committee of Nanjing Drum Tower Hospital and was performed in accordance with the ethical standards of the 2013 Declaration of Helsinki.

## Results

Deep tissue cultures obtained during the surgery were positive in 7 patients, revealing the presence of *Staphylococcus aureus* (*n* = 3), *Staphylococcus epidermidis* (*n* = 1), *Pseudomonas aeruginosa* (*n* = 1), *Acinetobacter baumannii* (*n* = 1), *Escherichia coli* (*n* = 1), *Enterobacter cloacae* (*n* = 1), and *Enterococcus avium* (*n* = 1) (Table 2). One patient whose culture was negative received vancomycin; other patients received sensitive antibiotics from the cultures.

All patients exhibited partial segmental bone defects [1] after debridement. The estimate volumes of bone defects were 75 cm^3^ (40–110 cm^3^). One patient had superficial wound breakdown early after surgery, which healed with local wound care. The remaining seven patients had no wound problems immediately after the surgery. All flaps healed uneventfully. The mean hospital stay was 21 (range, 14 to 31) days.

**Fig. 2:**
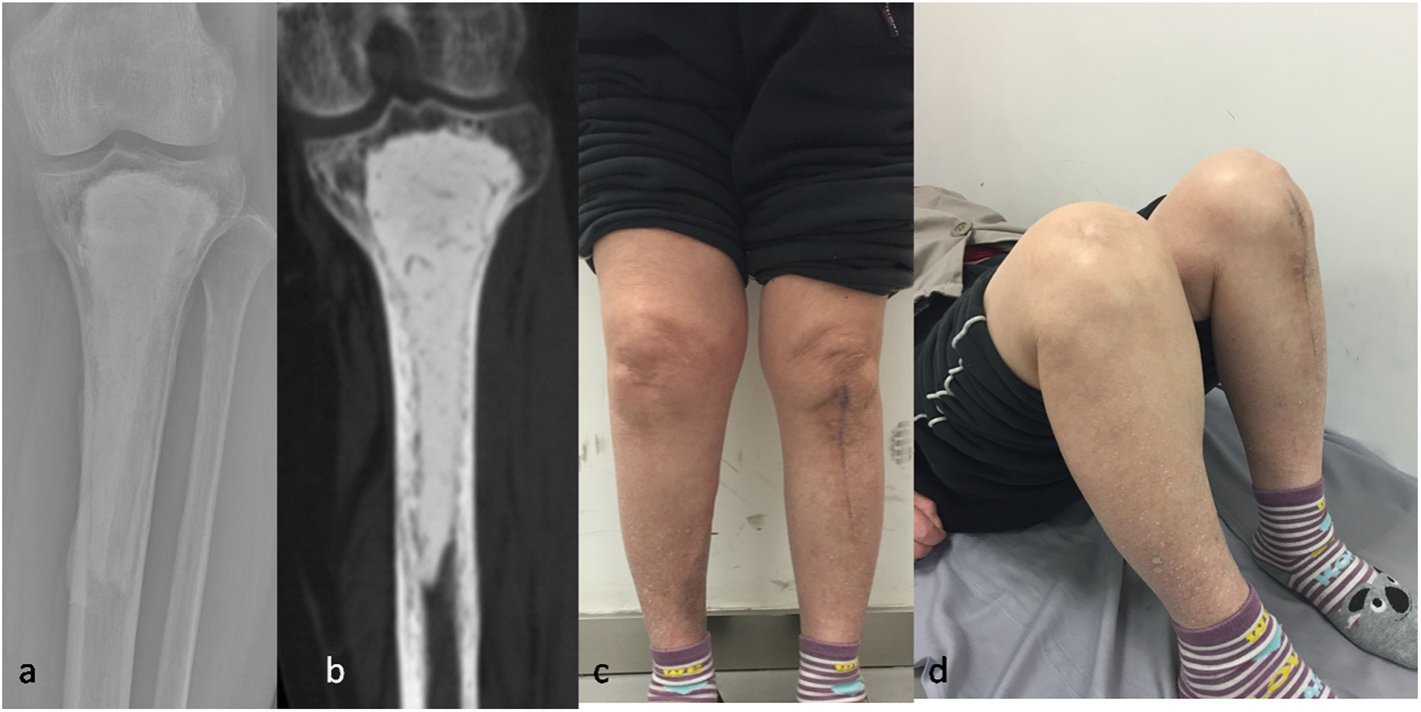
**a**-**b** At the 3-year follow-up, no excessive bone loss was observed on X-ray film and CT scan. **c** The patient achieved complete weight-bearing ability without pain; no sign of recurrence of the infection was observed. **d** The patient exhibited normal range of motion of the affected knee

At a mean of 2 years (6 months to 4 years) follow-up, all patients had the cement spacer in place. No patient had radiographic evidence of excessive bone loss (Fig. 2). The patients had no or occasional mild pain and did not use pain medication on a daily basis. Patients exhibited complete weight-bearing mobility and were able to perform their basic daily activities (Fig. 2), with the exception of one patient who used a crutch due to a spinal cord injury associated with spinal fracture. No case had any sign of recurrence of the osteomyelitis; no fractures were noted at the last follow-up (Fig. 2).

## Discussion

The management of chronic osteomyelitis is complicated and relies on a multidisciplinary approach, consisting of infection control with radical debridement, bone stabilization in the case of non-union or bone segment excision, local and systemic antibiotic therapy, dead space and wound management and a bone graft of non-united bone or large bone defects [13]. The main goals are elimination of infection and promotion of bone union. In the present study, all patients with osteomyelitis planned to receive a two-stage management procedure in our centre. The first stage involved radical debridement and insertion of an ACS into the bone defect. After debridement, all patients’ bones were relatively stable, and no stabilization was needed. Soft tissue was repaired in two patients. The second stage involving cement spacer removal and bone grafting was planned 6 to 8 weeks later. However, all of the patients did not receive the second stage reconstruction and retained the bone cement. At the last follow-up, these patients appeared to function well without necessarily requiring further surgical intervention.

The long-term use of cement spacers have been reported in periprosthetic joint infections and diabetic feet [5, 6, 8–11]. Choi et al. [6] reported eighteen patients with periprosthetic joint infection (11 hips and 7 knees) treated by prosthetic articulating spacers who retained their spacers and were followed up an average of 43.8 months (range, 13 to 78 months). Their study suggested that a proportion of patients with unplanned retention of prosthetic spacers appear to function well up to 6 years. Haddad et al. [8] used ACS as a definitive treatment for a patient with post-arthroscopy shoulder destructive osteomyelitis. The patient maintained excellent function with no radiological signs of wear or loosening 4 years after surgery. Ferrao et al. [9] reported using ACS as definitive management for postoperative ankle infection in nine patients. The average time of cement spacer retention was 20.1 months (range, 6 to 62 months). At the final follow-up, seven patients still retained their cement spacer, and two had below knee amputations because of delayed complications. All patients with a retained cement spacer were mobile and able to perform daily activities with minimal discomfort. Melamed et al. [10] reported osteomyelitis and associated severe infection of forefoot joints in 20 patients with diabetic neuropathy. Extensive debridement and ACS was used in these patients. Of these patients, 91.3 % healed, and two required toe amputation. Cement spacers permanently remained in 10 patients, were removed with arthrodesis in six patients, and removed without arthrodesis in five patients. The author concluded that severe infection associated with osteomyelitis of the foot in diabetic patients was successfully treated with extensive debridement and the use of ACS.

To our knowledge, no reports about the long-term use of ACS in patients with osteomyelitis in long bone are available. Our study was the first report the long-term use of ACS in patients with osteomyelitis in long bones. Consistent with previous periprosthetic joint infection reports, our study also revealed satisfactory clinical outcomes. No patient had evidence of excessive bone loss radiographically. The patients exhibited complete weight-bearing mobility and were able to perform their daily activities. No case exhibited any sign of recurrence of the osteomyelitis; and no fractures occurred.

Bone resorption is a potential issue associated with the retention of a temporary spacer. Recently, Regis et al. [5] reported one case with 6 years of follow-up for a retained ACS for the management of chronically infected total hip replacement. Although the patient recovered a good range of motion and was able to walk pain free with assisted weight bearing, a slowly progressive resorption of the cortical femur around the stem and fatigue fracture of the stem of the spacer (at 2 years) were observed. Therefore, these researchers suggested that prolonged spacer implantation was not appropriate as a permanent solution for septic hip replacement, and careful periodic monitoring was required. In the present study, no obvious bone loss was observed radiographically. Two factors potentially account for this observation. First, the duration of follow-up was relatively short in our study. Second, the retained cement spacer in the joint was mobilisable, whereas the cement spacer in our study was immobilisable.

Another common concern about the retained ACS is that the long-term exposure to low dose antibiotics from bone cements in patients is strongly related to the emerging threat of antibiotic resistance [14]. Anagnostakos et al. [15] evaluated this problem by examining 18 chains of antibiotic-loaded beads that were implanted for the treatment of orthopaedic infections. In 4 cases, persistent bacterial growth was noted on the beads. The emergence of a gentamicin-resistant *S. epidermidis* strain was noted in one case despite the fact that preoperative samples of *S. epidermidis* from this patient were susceptible to the antibiotic. Their study reveals that the persistence of bacterial growth on bone cement remains a hazardous problem and that adherence of bacteria to cement can lead to the emergence of antibiotic-resistant bacteria, possibly resulting in clinical recurrence of the infection. However, we believe that inadequate debridement is the main cause for recurrence. The present study demonstrated satisfactory infection control (100 %) with this type of spacer, which is consistent with previous periprosthetic joint infection reports [4, 6–9]. This result may be attributed to the thorough debridement.

In summary, we report on the fate of retained cement spacers in the management of osteomyelitis. Although retention of temporary spacers is not the standard management and would not be appropriate in all cases of osteomyelitis, our study suggests that some of these retained spacers may have good clinical outcomes due to satisfactory infection control and limb function. A further prospective study is required to identify the factors that contribute to the successful application of this technique.
